# Comparative Study of Electrical Stimulation of the Heart with VDD and DDD Pacemakers as to the Evolution to Atrial Fibrillation

**DOI:** 10.21470/1678-9741-2017-0505

**Published:** 2017

**Authors:** Nelson Leonardo Kerdahi Leite de Campos, Rubens Ramos de Andrade, Marcello Laneza Fellicio, Antônio Sergio Martins, André Monti Garzesi, Leonardo Rufino Garcia, Tassya Bueno Takeda

**Affiliations:** 1 Faculdade de Medicina de Botucatu (Unesp), Botucatu, SP, Brazil.

**Keywords:** Pacemaker, Artificial, Atrial Fibrillation, Atrioventricular Block

## Abstract

**Introduction:**

The pacemaker implantation VDD is considered simpler, faster, less expensive and causes fewer complications compared to DDD. However, the VDD pacemaker has not been widely used in many centers, perhaps for fear of dysfunction of the sinus node and the reduction of atrial sensitivity by the pacemaker during follow-up after implantation.

**Objective:**

To compare patients with DDD and VDD pacemakers regarding the evolution of chronic atrial fibrillation (AF) and length of stay outside this postoperative arrhythmia.

**Methods:**

It was included 158 patients with dual chamber pacemakers, 48 DDD and 110 VDD. Follow-up period: between January 1, 1999 and December 31, 2015. The mean follow-up of patients with DDD was 5.35 years and the VDD, 4.74 years. The percentage of each group (DDD and VDD) which evolved to AF during follow-up was assessed. Also, it was made an actuarial study with the respective curves indicating the time free from AF for each group. Patients were classified according to the diagnosis that led to pacemaker implantation and the degree of heart failure.

**Results:**

The percentage of patients who developed AF was higher in DDD group (10.42%) than in VDD group (6.36%), but without statistical significance. Patients with DDD and VDD remained free of AF for similar period.

**Conclusion:**

Considering the results, the VDD pacemaker continues to be a good option to the DDD for routine use in cases properly indicated.

**Table:** 

Abbreviations, acronyms & symbols	
AF	= Atrial fibrillation

## INTRODUCTION

In electric cardiac stimulation, the dual chamber pacemaker is widely used, especially in DDD mode, indicated in cases of atrioventricular heart block, among others. This type of stimulation offers many clinical advantages as it can maintain synchronization between atriums and ventricles leading to hemodynamic gain during the cardiac cycle and improves cardiac output. This type of pacing is made by the implantation of two leads, one in the right atrium and the other in the right ventricle, coupled to a dual-chamber generator, each lead may sense or stimulate the heart, thus working on demand mode according to the cardiac requirements.

The VDD mode is an alternative kind of stimulation of dual chamber pacemakers generators. This type of cardiac stimulation can be used when the sinus node is normally functioning^[1]^. In this case, only one lead is implanted, where its end remains in the right ventricle's internal wall for stimulate and sense at this location. This same lead has a proximal pole that is positioned within the right atrium and at this point is only able to sense the heartbeats.

The ideal leads' position in both types of pacemakers is achieved by positioning them with fluoroscopy aid in addition to reading the appropriate electronic measures at the chosen location.

According to Eberhardt et al.^[2]^, the use the VDD pacemaker allows to implant in a shorter time, with less use of fluoroscopy and fewer complications compared to DDD. Moreover, the VDD pacemaker has less cost than DDD, considering material, hospital and medical fees^[3]^. Gonçalves et al.^[4]^ evaluated VDD implantation and concluded that this mode of stimulation permits clinical and hemodynamic improvement, indicating this pacemaker in all patients with atrial stability and competence.

The implant of VDD pacemaker is not widely made in medical practice, despite the advantages mentioned above. One hypothesis for this is the sinus node dysfunction fear and decrease of the sensitivity by the pacemaker after its implantation, during follow-up of these patients^[5]^.

Pakarinen and Toivonen^[6]^ state that careful evaluation of the clinical history, electrocardiogram and adequate radiographs are sufficient to select elderly candidates for VDD pacemaker implantation and thus, sinus dysfunction rarely occurs under these conditions.

Although some references show that this type of pacemaker has not been used frequently^[7]^, we routinely implant VDD pacemakers in daily clinical practice without finding significant complications inherent in this procedure; however, we observed that some patients developed atrial fibrillation (AF) after implantation, which we also observed in DDD implants.

Therefore, it is appropriate to question if the presence of a lead in the right atrial wall of DDD pacemakers would increase the risk of AF induction, or, on the other hand, the atrial pacing lead would promote more protection against this arrhythmia, as some reports suggested^[8]^.

The objective of this study was to compare patients with DDD and VDD pacemakers with respect to the evolution towards chronic AF and the time free of this arrhythmia in postoperative period by evaluation in Pacemaker Clinic of Faculdade de Medicina de Botucatu - Unesp.

## METHODS

This study included patients undergoing cardiac pacemaker implantation between January 1, 1999 and December 12, 2014, attended at the Outpatient Hospital of Faculdade de Medicina de Botucatu. Outpatient follow-up was considered the period between January 1, 1999 and December 31, 2015.

### Approval by CEP

This study was approved by the Ethics and Research Committee, with CAAE: 50099715.2.0000.5411 with sound number: 1314,841.

### Patients

From 625 we included 158 patients with dual-chamber pacemaker with 48 DDD and 110 VDD. It was accomplished a gender distribution, diagnosis, functional class of heart failure.

### Diagnostics for Implants Indication

The diagnostics that led indications of pacemakers implants are listed in Table 1.

**Table 1:** Distribution in each group: number of patients studied; sex; previous diagnosis to the implant; functional class of heart failure; mean follow-up of patients in months (MFM).

		DDD (number of patients)	VDD (number of patients)	*P*
Number of patients		48	110	
Men		24	57	0.9703
Women		24	53	0.9703
Prior diagnosis	1^st^ AVB	1	3	1
	2^nd^ AVB I	2	7	0.8613
	2^nd^ AVB II	35	97	0.03182
	CSH	8	0	_
	RB1AVL	1	1	_
	LBBB	0	2	_
	BTS	1	0	_
Functional class	I	11	20	0.6373
	II	29	70	0.8368
	III	7	19	0.8524
	IV	1	1	_
MFM (months) standard deviation		64.16 (or 5.35 years) 45.98	56.88 (or 4.74 years) 45.81	0.3895

1^st^ AVB= first-degree atrioventricular block; 2^nd^ AVB I=second degree atrioventricular block – Mobitz 1; 2^nd^ AVB II=atrioventricular block second degree – Mobitz 2; CSH=carotid sinus hypersensitivity; RB1AVL=right bundle branch block + first-degree atrioventricular block + left anterior-superior division of the left bundle branch block; LBBB=left bundle branch block; BTS=brady-tachy syndrome. Functional class=The New York Heart Association functional classification (I, II, III and IV heart failure). *P* value when comparing the parameters of DDD and VDD. It was considered a significance level of *P*<0.05.

### Mean Time of Patient Monitoring

Average times of follow-ups of patients undergoing pacemaker implants are indicated in Table 1. The total time in years follow-up added all patients of each type of pacemaker was 256.66 years to DDD and 521.41 pacemaker years to VDD. Outpatient follow-up was considered the period between January 1, 1999 and December 31, 2015, the shortest follow-up time of 2 months and the longest of 13 years and 11 months.

### Exclusion Criteria

We excluded patients with third degree atrioventricular block and sinus node disease in both groups. We also excluded cases where doubts diagnostic occurs, information were insufficient and data were not fully reliable.

### Groups

Patients were classified into 2 groups: Group 1, patients with DDD pacemakers; Group 2, patients with VDD pacemakers.

### Atrial Fibrillation

In both groups, patients who developed post-implantation AF were separated and the time for development of AF after surgery was measured in each of them. Only patients who entered in AF at least 2 months after surgery were included.

### Comparison between Number and Percentage of Each Group for Evolution to AF

Comparisons between DDD and VDD groups were made regarding the number of patients and percentage that evolved AF within each group.

### Actuarial Study

Free time of AF was evaluated by the curves and actuarial study in both groups.

### Statistical Study

One proportion test was performed to verify if there was statistical difference between the proportions of variables such as gender, previous diagnosis and functional class in both groups.

For the time variable, a normality test was performed and the data showed an asymmetrical distribution. Thus, adjusted a generalized linear model with gamma distribution to check whether there was difference between the groups with respect to time (months) of follow-up

For the actuarial study we used the Statistical Calculations for Windows V. 1.8, developed by Dr. Domingo Braile and Dr. Moacir Fernandes de Godoy and implemented in Power Builder 6.6 Sc. Djalma Domingos Silva. The construction of actuarial curves was made in Microsoft Excel program.

## RESULTS

### Number and Percentage of Patients who Developed AF

Table 2 shows the number of patients and the percentage that has evolved to AF in both groups during the period considered in the study.

**Table 2:** Distribution in number (N) and percentage (%) of each type of pacemaker which remained in sinus rhythm and which progress to atrial fibrillation (AF) in groups DDD and VDD.

	DDD		VDD		*P*
Number of patients	48		110		
	N	%	N	%	
In sinus rhythm	43	89.58	103	93.64	0.5769
Evolution to AF	5	10.42	7	6.36	0.5769

It was considered a significance level of *P*<0.05. AF=atrial fibrillation

### Actuarial Calculations and Curves

**Fig. 1:**
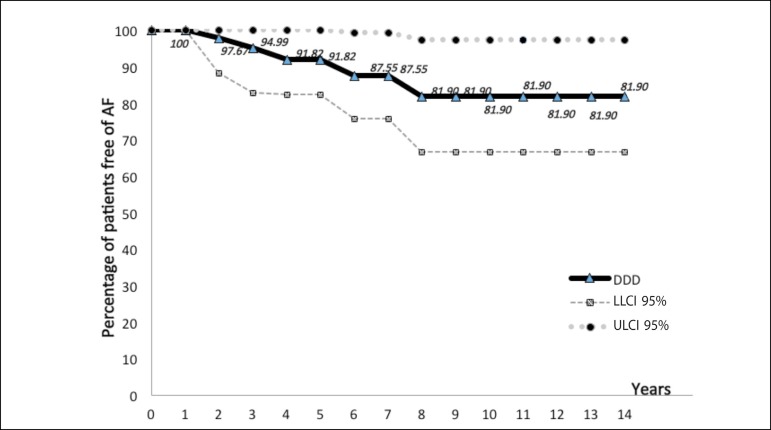
Actuarial curve of patients with dual chamber pacemaker DDD, showing the proportion of patients free from AF (ordinate) - expressed values close to the corresponding points on the curve for the years elapsed (abscissa). They also observed the curves of LLCI 95% (lower limit confidence interval 95%) and ULCI 95% (upper limit of confidence interval 95%).

**Fig. 2:**
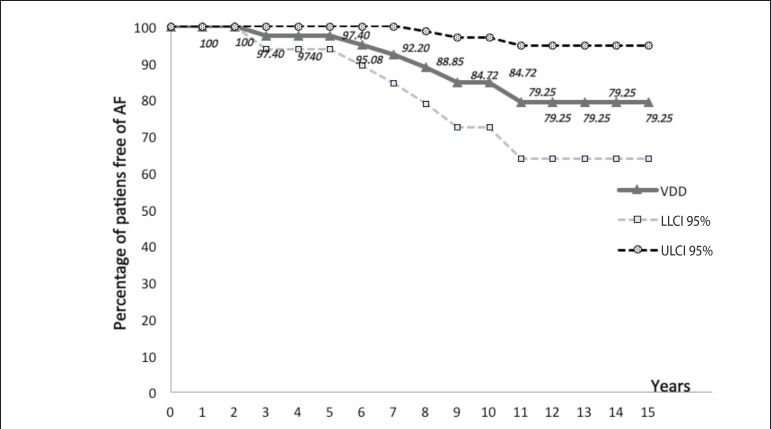
Actuarial curve of patients with dual chamber pacemaker VDD, showing the proportion of patients free from AF (ordinate) - expressed values close to the corresponding points on the curve for the years elapsed (abscissa). They also observed the curves of LLCI 95% (lower limit confidence interval 95%) and ULCI 95% (upper limit of confidence interval 95%).

**Fig. 3:**
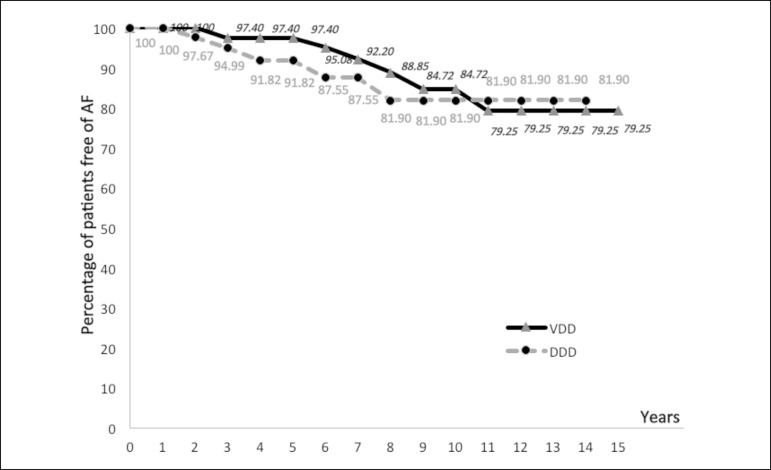
Actuarial curve of patients with dual chamber pacemaker VDD and DDD, showing the proportion of patients free from atrial fibrillation-AF (ordinate) - expressed values close to the corresponding points on the curve for the years elapsed (abscissa).

The actuarial study comparing the incidence of AF in patients with dual chamber pacemaker DDD and VDD is shown in Figures 1, 2 and 3 and Tables 3 and 4.

**Table 3:** Actuarial calculations of DDD group.

Years	PFE%	SE%	LLCI 95%	ULCI 95%
1	100	0.00	100	100
2	97.67	2.30	93.10	100
3	94.99	3.46	88.21	100
4	91.82	4.57	82.27	100
5	91.82	4.57	82.27	100
6	87.55	6.03	75.73	99.37
7	87.55	6.03	75.73	99.37
8	81.90	7.85	66.51	97.29
9	81.90	7.85	66.51	97.29
10	81.90	7.85	66.51	97.29
11	81.90	7.85	66.51	97.29
12	81.90	7.85	66.51	97.29
13	81.90	7.85	66.51	97.29
14	81.90	7.85	66.51	97.29

PFE%= proportion of patients free of events (atrial fibrillation); SE%= standard error; LLCI 95%= lower limit of confidence interval 95%; ULCI 95%= upper limit of confidence interval 95%

**Table 4:** Actuarial calculations of VDD group.

Years	PFE%	SE%	LLCI 95%	ULCI 95%
1	100	0.00	100	100
2	100	0.00	100	100
3	97.40	1.81	93.85	100
4	97.40	1.81	93.85	100
5	97.40	1.81	93.85	100
6	95.08	2.89	89.41	100
7	92.20	3.99	84.38	100
8	88.85	5.06	78.93	98.77
9	84.72	6.29	72.39	97.05
10	84.72	6.29	72.39	97.05
11	79.25	7.91	63.74	94.76
12	79.25	7.91	63.74	94.76
13	79.25	7.91	63.74	94.76
14	79.25	7.91	63.74	94.76
15	79.25	7.91	63.74	94.76

PFE%= proportion of patients free of events (atrial fibrillation); SE%= standard error; LLCI 95%= lower limit of confidence interval 95%; ULCI 95%= upper limit of confidence interval 95%

## DISCUSSION

The concept VDD pacemaker was designed in 1973, but only became commercially available in the 80s^[9]^. Mond et al.^[7]^, in a study performed in 2005 by Cardiac Pacing and ICD Survey, which compared the use of pacemakers in 43 countries, Brazil ranked fourth in a VDD use in percentage values, surpassed by Spain (20%), Japan (18%) and Italy (11%). According to this study, in Brazil 53% of pacemakers used was DDD (R), 34% VVI (R), 9% VDD, 4% biventricular and less than 1% AAI (R). During the period considered in this work we routinely use the VDD pacemaker. It can be seen that the VDD group is bigger (110 patients) than DDD group (48 patients).

Eberhardt et al.^[2]^ conducted a retrospective study in 1884 patients who received unicameral (VVI, 610 patients) and bicameral pacemakers (VDD, 371 patients, and DDD, 903 patients). Surgeon's experience was considered in three levels according to the number of previous implants made before the study (low: less than 50 implants; middle: between 50 and 100 implants, and high: over 100 implants). They observed that the DDD pacemaker requires longer use of fluoroscopy when implanted by surgeons with low or middle levels of experience, while this time is substantially reduced with higher level of experience. The risk of complications in procedures performed by surgeons with low and middle levels of experience were significantly higher for implants of DDD pacemakers, compared to VDD or VVI. However, this difference disappears when operations were performed by surgeons with better expertise.

Wiegand et al.^[3]^ conducted a study in order to compare costs between the implants of DDD and VDD pacemakers. The authors state that using VDD pacemaker, the overall cost of an uncomplicated implant (including prostheses) can be reduced by 9% (EUR 518 per patient in this study). They concluded therefore that the treatment with VDD pacemaker promotes a significant cost reduction compared to DDD, without loss of efficacy. Therefore, the single lead used in VDD pacemaker can promote a satisfactory cost-benefit ratio in the treatment of patients with high-grade atrioventricular block with normal sinus node function, when considering the use of dual-chamber pacemaker.

Given these reports, it is curious the fact that the VDD pacemaker is less used than would be expected. Schaer et al.^[10]^ claim that, in clinical practice, VDD pacemaker has an excellent performance in atrioventricular block patients with a presumed normal function of the sinus node, with very low incidence of need for review by loss of atrial sensitivity (2%) and rarely requiring reintervention for DDD pacemaker implant due sinus node dysfunction (1%). According to the authors, the main reason for reprogramming the VDD pacemaker to VVI is the beginning of permanent AF.

Marchandise et al.^[5]^, in a prospective, non-randomized study, compared patients with symptomatic atrioventricular block who received VDD and DDD pacemakers. According to the authors, despite the several advantages offered by the pacemaker VDD, the main disadvantage is that patients develop a greater loss of atrial detection compared to DDD. However, it was not found other differences between the two types of pacemaker that could lead to clinical impact on the incidence of AF, myocardial infarction, dilated cardiomyopathy or mortality.

We have not included in our study patients with a previous diagnosis of sinus node disease and third degree atrioventricular blocking, intending to become more homogeneous groups, considering 107 cases of sick sinus syndrome in DDD and 0 in VDD group. In addition, the number of patients with third degree atrioventricular block was 84 in DDD and 276 in VDD group. Considering this relevant numerical difference of third degree atrioventricular blocking, we also opted for the exclusion of patients with this type of blocking. This decision resulted in a decrease in the number of patients studied, but we believe that the comparison between the groups was best suited.

Comparing VDD and DDD groups we found no differences with statistical significance between sex and functional class or follow-up average time. Regarding the pacemaker indication, we observed that most of cases was second degree atrioventricular block Mobitz I and II, being that in the VDD group the second degree atrioventricular block Mobitz II was significantly more present (Table 1).

Although the percentage of patients who developed AF during the period studied was lower in VDD group (6.36%) than in DDD group (10.42%), there was no statistical significant difference between them (Table 2), corroborating the results of Marchandise et al.^[5]^, which also found no differences in this sense, even though they have found greater loss of atrial detection in VDD pacemaker.

Wiegand et al.^[11]^ found that the occurrence of sinus node disease is rare in patients with isolated atrioventricular block and the incidence of atrial tachyarrhythmia in patients undergoing pacemaker implantation DDD and VDD were similar.

With the actuarial study we observed that the two actuarial curves were close all the time. In the early years, the curve of VDD group is positioned slightly above DDD, but this position is reversed in the last years of follow-up. Thus, we believe that the closeness of both curves and the actuarial calculations do not demonstrate a relevant difference in length of time which patients with VDD and DDD pacemakers were free of AF (Figures 1, 2 and 3, Tables 3 and 4).

According to Pakarinen and Toivonen^[6]^, the VDD pacemaker should not be used in patients with paroxysmal AF and increased size of heart due to higher probability of loss of atrial function with progressive evolution to AF.

The results obtained combined with reports in the literature (lower incidence of complications, shorter time of use of fluoroscopy for the implant, lower cost, simpler system with easier implantation) leads us to believe that VDD pacemaker remains a good option when duly indicated. In selecting patients which would receive this type of pacemaker (VDD) is needed careful evaluation of clinical history with electrocardiographic and radiological appropriate study, thus, in this case, the loss of atrial detection and/or AF rarely will develop.

## CONCLUSION

The absence of a lead in the right atrium, in the case of VDD pacemakers, besides promoting fewer complications, simplifying the surgery and having a lower cost, seems not interfere in the development of AF when compared to DDD pacemaker, without loss of its efficiency.

**Table:** 

Authors' roles & responsibilities	
NLKLC	Conception and design study; realization of operations and/or trials; analysis and/or data interpretation; manuscript redaction or critical review of its content; final manuscript approval
RRA	Realization of operations and/or trials; final manuscript approval
MLF	Realization of operations and/or trials; final manuscript approval
ASM	Realization of operations and/or trials; final manuscript approval
AMG	Realization of operations and/or trials; final manuscript approval
LRG	Realization of operations and/or trials; final manuscript approval
TBT	Realization of operations and/or trials; final manuscript approval
